# Subconjunctival dexamethasone implant (Ozurdex^®^) in the management of refractory Non-Infectious anterior scleritis

**DOI:** 10.1186/s12348-025-00494-6

**Published:** 2025-05-06

**Authors:** Battuya Ganbold, Ba Trung Nguyen, Jia-Horung Hung, Azadeh Mobasserian, Zheng Xian Thng, Hashem Ghoraba, Negin Yavari, Dalia El Feky, Cigdem Yasar, Aim-On Saengsirinavin, Xiaoyan Zhang, Frances Andrea Anover, S. Saeed Mohammadi, Ngoc Tuong, Trong Than, Anadi Khatri, Osama Elaraby, Amir Akhavanrezayat, Ankur Sudhir Gupta, Woong Sun Yoo, Quan Dong Nguyen, Christopher Or

**Affiliations:** 1 Spencer Center for Vision Research, Byers Eye Institute at Stanford University, 2452 Watson Court, Suite 200, Palo Alto, CA 94303 USA; 2 Bolor Melmii Eye Hospital, Ulaanbaatar, Mongolia; 3 Department of Ophthalmology, Faculty of Medicine, Tanta University, Tanta, Egypt

**Keywords:** Non-infectious anterior scleritis, Subconjunctival dexamethasone implant, Ozurdex^®^

## Abstract

**Objective:**

To report a case series of non-infectious anterior scleritis resistant to multiple lines of conventional therapies which were eventually successfully treated with off-label subconjunctival dexamethasone implant (Ozurdex^®^) injection (SDI).

**Methods:**

A retrospective case series of 4 patients (6 eyes).

**Results:**

In the index case series, the patients had a mean age of 57.2 years (range 36 to 82 years, SD 19.2 years) with 50% being female. Two patients had underlying autoimmune diseases: rheumatoid arthritis (*n* = 1), and granulomatosis with polyangiitis (GPA) (*n* = 1). The other patients were diagnosed with idiopathic anterior scleritis after extensive systemic investigations (*n* = 2). The mean follow-up duration and the mean number of concomitant therapies prior to SDI was 27 (SD 17.7) months and 2 (SD 0.81), respectively. In all patients, symptom resolution and significant improvement in disease activity were achieved after SDI, persisting for an extended period following the resorption of the implant. No scleral melt, infection or ocular hypertension were noted following SDI.

**Conclusion:**

SDI may be a safe and effective therapeutic option for resistant non-infectious anterior scleritis.

## Introduction

Non-infectious scleritis is an inflammatory condition of the sclera and a potentially sight-threatening disease. Clinical presentations vary based on anatomical location and severity. Watson and Hayreh’s classification categorizes the condition into anterior and posterior scleritis, with anterior scleritis further divided into diffuse scleritis, nodular scleritis, necrotizing with inflammation, and necrotizing without inflammation (scleromalacia perforans) [1]. This classification is widely accepted and has therapeutic and prognostic implications. Diffuse anterior scleritis is the most common, followed by anterior nodular, necrotizing, and posterior [2, 3]. Notably, necrotizing scleritis, despite its relative rarity, manifests as the predominant subtype of scleritis associated with a decrease in vision [3]. Anterior scleritis typically manifests with ocular erythema and tenderness, while posterior scleritis may present with blurred vision, with or without pain. Rheumatoid arthritis and granulomatosis with polyangiitis are the two most common autoimmune diseases associated with scleritis [4–6]. Less commonly implicated systemic conditions include relapsing polychondritis, polyarteritis nodosa, spondylo arthropathies, and systemic lupus erythematosus [7, 8].

Long-term management of non-infectious scleritis can be challenging due to high recurrence rates and severe adverse side effects associated with its therapy. Topical corticosteroids and oral nonsteroidal anti-inflammatory drugs (NSAIDs) have been considered the mainstay therapy for mild to moderate cases [9, 10], with oral corticosteroids being administered in cases of inadequate response [8, 11]. Even though highly efficacious in the acute setting, significant longer-term systemic and ocular side effects preclude corticosteroids as maintenance therapy at doses above 7.5mg daily [8, 12, 13]. Immunosuppressants are considered next in the stepladder approach when patients are refractory with the aforementioned treatments, have multiple recurrences during corticosteroid taper or are intolerable to the side effects of corticosteroids. Biologic agents, including anti-tumor necrosis factor (TNF) and anti-CD 20 monoclonal antibody (i.e., rituximab), may be used for severe or refractory cases [3, 10, 14, 15]. Local corticosteroid therapy, such as subconjunctival or sub-tenon corticosteroid injection has demonstrated success in non-infectious, non-necrotizing anterior scleritis [16–18]. Although the published cases showed rapid resolution of inflammation with a good safety profile, subconjunctival corticosteroid are used with caution because of concerns regarding the risk of scleral thinning or perforation [16, 19].

Dexamethasone intravitreal implant (DEX implant, Ozurdex^®^, Allegan Inc, Irvine, CA) is a biodegradable rod-shaped dexamethasone-releasing implant which is approved by the United States Food and Drug Administration for the treatment of intermediate, posterior and panuveitis, diabetic macular edema, and macular edema secondary to retinal vein occlusion [20]. While originally designed for intravitreal injection, the DEX implant has been safely and effectively administered subconjunctivally for various indications, including scleritis and prevention of graft rejection post-penetrating keratoplasty [21, 22].

Herein, we present a case series comprising three instances of noninfectious scleritis and one case of peripheral ulcerative keratitis (PUK) successfully managed with subconjunctival dexamethasone implant (SDI).

## Case 1

A 60-year-old Hispanic woman with a past medical history of type 2 diabetes mellitus and rheumatoid arthritis was presented to a tertiary ophthalmic referral center with persistent eye pain and redness in both eyes. She was diagnosed with rheumatoid arthritis (RA) at the age of 44 which has been difficult to manage. From 2008 to 2022, she was trialed on a diverse range of medications, including antimetabolites such as methotrexate, leflunomide, and azathioprine, alongside biologic therapies such as adalimumab, etanercept, abatacept, certolizumab, rituximab, and tofacitinib, either individually or in combination. However, all medications were discontinued due to either ineffectiveness or intolerance. She continues to have severe diffuse anterior scleritis during this period necessitating treatment with chronic systemic and topical steroids. In addition, the rheumatologist initiated the patient on cyclophosphamide due to persistent systemic inflammation. The patient reported a three-year history of anterior scleritis and confirmed continuous use of oral and topical steroids (prednisone) throughout this period without interruption during cyclophosphamide treatment.

**Fig. 1:**
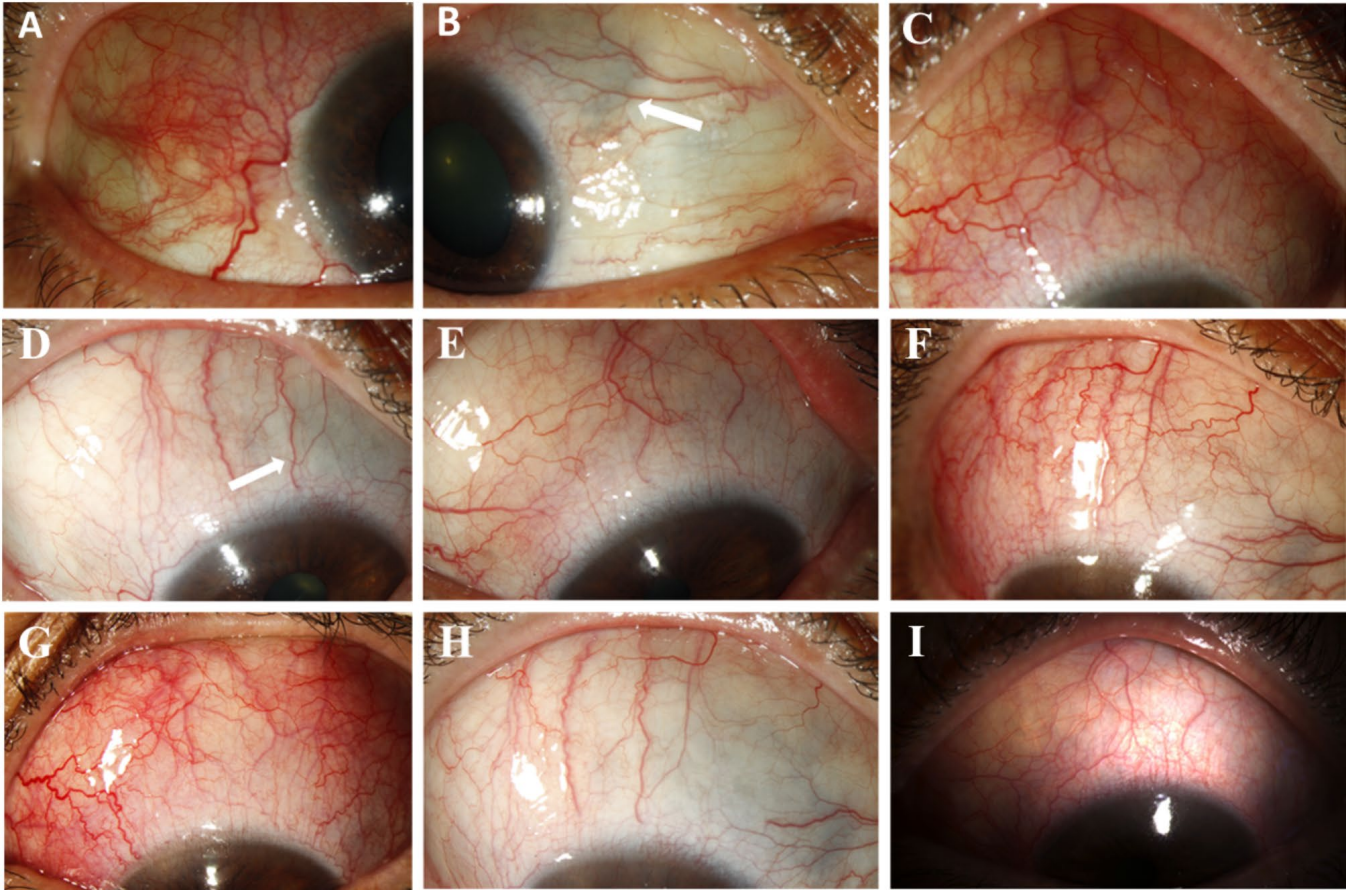
Clinical photographs of Case 1. Slit-lamp photographs obtained at the initial examination of the 60-year-old female patient. There was superotemporal scleritis in her right eye **(A)**, superonasal scleral thinning in her right eye **(B**,** arrow)**, and injection of the superior sclera in her left eye **(C)**. The scleral injection improved following systemic and periocular steroid (TA) treatment **(D).** The superonasal scleral thinning in her right eye remains stable **(E**,** arrow)**. The scleritis also improved in her left eye after systemic and periocular steroid (TA) treatment. The anterior scleritis recurred 5 months following the initial visit in her right eye (before SDI) **(F)**, and left eye (before SDI) **(G)**. Six months after SDI, the sclera was quiet in her right eye **(H)**. Six months after SDI, mild superior scleral injection recurrence was seen in her left eye **(I)**

On her initial presentation, her best corrected visual acuity (BCVA) was 20/25 in right eye (OD) and 20/30 in left eye (OS), with normal intraocular pressures (IOP) bilaterally. Her eye ocular examination demonstrated 3 + injection in the superotemporal quadrant (Fig. 1A) and scleral thinning in the superonasal quadrant in OD (Fig. 1B). 3 + conjunctival injection was present in the superonasal and superior quadrants in OS (Fig. 1C). Funduscopic examination, wide angle fundus photography, fluorescein angiography (FA), and spectral-domain optical coherence tomography (SD-OCT) were otherwise unremarkable. Systemic workup was unrevealing except for a positive rheumatoid factor (RF), elevated C-reactive protein (CRP) of 1.2 mg/dl and an elevated hemoglobin A1c (HbA1C) of 8.7%.

At her initial visit, we increased the amount of oral prednisone to 40 mg per day with a tapering schedule, and performed a subtenon triamcinolone acetonide injection (20 mg, 0.5 ml) in her left eye. Subsequently, the eye redness and pain improved in both eyes (Fig. 1D, E). Unfortunately, anterior scleritis recurred (OD < OS) 5 months after her initial visit (Fig. 1F, G). SDI was then administered to the superior quadrant in both eyes. At her most recent visit, there was no pain, and the sclera was quiet in her right eye (Fig. 1H) with mild injection in her left eye (Fig. 1I).

Her VA was unchanged. The IOP was 18 and 15 mmHg. She remained stable on prednisone (5 mg) along with prophylactic topical timolol (0.5% twice daily).

## Case 2

A 36-year-old Hispanic man was referred to a tertiary ophthalmic referral center due to progressively worsening blurry vision and recurrent eye redness and pain in the left eye (OS).

He had been diagnosed with granulomatosis with polyangiitis (GPA) at the age of 34, presenting with skin rash, left-side joint pains, and a positive antibody to Proteinase 3 (PR3). Histology from skin biopsy showed leukocytoclastic vasculitis with granulomatous infiltrate, confirming the diagnosis of GPA. He also had a history of central serous chorioretinopathy (CSCR) (OS). Initial treatment included oral prednisone (60 mg per day), rituximab (375 mg/m^2^ monthly) and mycophenolate mofetil (1000 mg twice daily). A year later, his disease flared requiring treatment with tofacitinib and subsequently, intravenous cyclophosphamide (750 mg) for 5 months. Symptoms of scleritis (OS) occurred then and were treated with daratumumab, rituximab, high-dose pulse steroid therapy for three days, and oral prednisolone (80 mg daily) for maintenance.

**Fig. 2:**
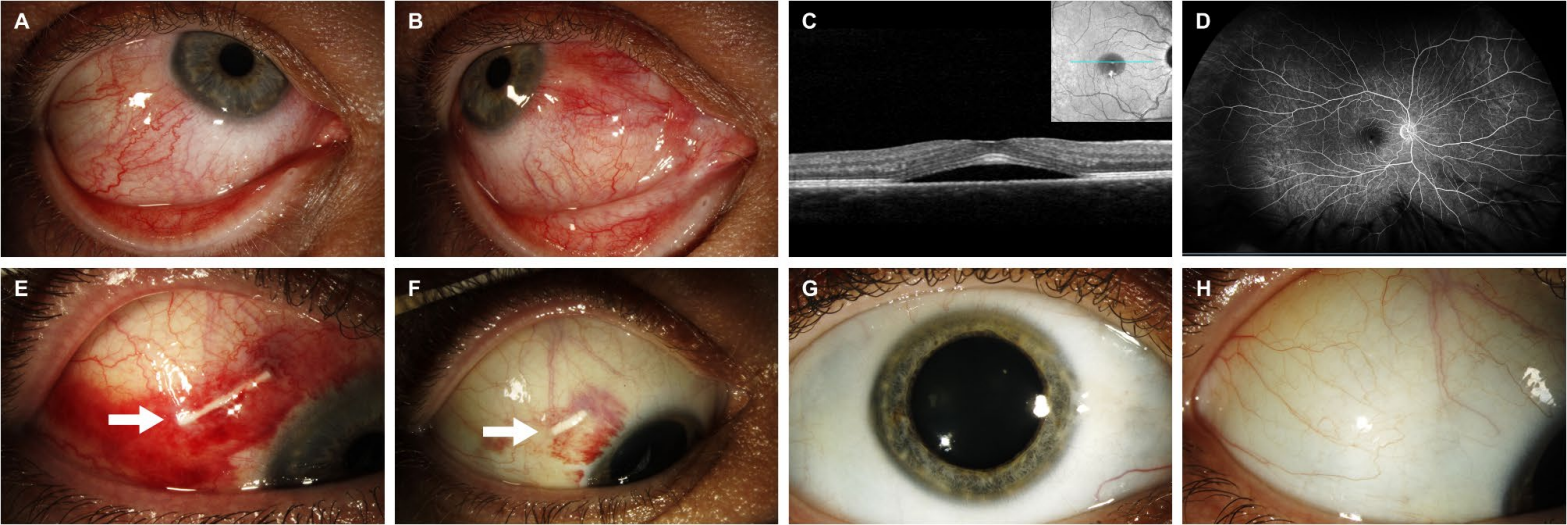
Clinical photographs of Case 2. Slit lamp photos of the right eye showed scleritis in the temporal **(A)** and nasal **(B)** quadrants. Spectral domain optical coherence tomography showed subretinal fluid with from CSCR **(C)**. Wide-field fluorescein angiography showed pinpoint leakage with leakage later in the macular region and a normal periphery at 5 min after dye injection **(D)**. Two days after SDI, the implant was observed to be in the subconjunctival space with accompanying subconjunctival hemorrhage **(E)**. The implant shortened 14 days later **(F)**. 17 months after the first visit, the sclera was quiet **(G)**, and the implant could not be observed **(H)**

The patient started to experience severe redness and pain in the right eye, and slit lamp examination showed severe scleral injection in the nasal and temporal sides, compatible with the diagnosis of scleritis (OD) (Fig. 2A **and B)**. Funduscopic examination showed CSCR (OD) (Fig. 2C **and D)**.

Initial evaluations, including liver function tests, creatinine, basic metabolic panel, HbA1c, angiotensin-converting enzyme, lysozyme, QuantiFERON, syphilis titers, anti-dsDNA, and anti-HIV antibody were within normal limits. Chest X-ray and MRI of the orbit were normal. Urinalysis showed hematuria. Complete blood count was remarkable for leukocytosis (at 16.3 K/µL) and decreased hemoglobin (at 12.9 g/dl), that had previously been low (around 9.0 g/dl) in the prior year.

SDI was administered to the superotemporal quadrant (Fig. 2E), eventually leading to significant improvement in symptoms. Two weeks later, the sclera was quiet with residual subconjunctival hemorrhage, and it is noted that the SDI shortened (Fig. 2F) and became invisible after 3 months. Repeated IOP measurements were normal post-injection. Fundus exam showed that the CSCR in the OD subsided without recurrence following SDI. Intravenous rituximab infusions continued every three months. At the last visit (17 months after the first visit), the sclera was quiet without recurrence of inflammation (Fig. 2G) and the implant was not visible (Fig. 2H). Visual acuity (OD) was 20/40 due to posterior subcapsular cataract formation.

## Case 3

**Fig. 3:**
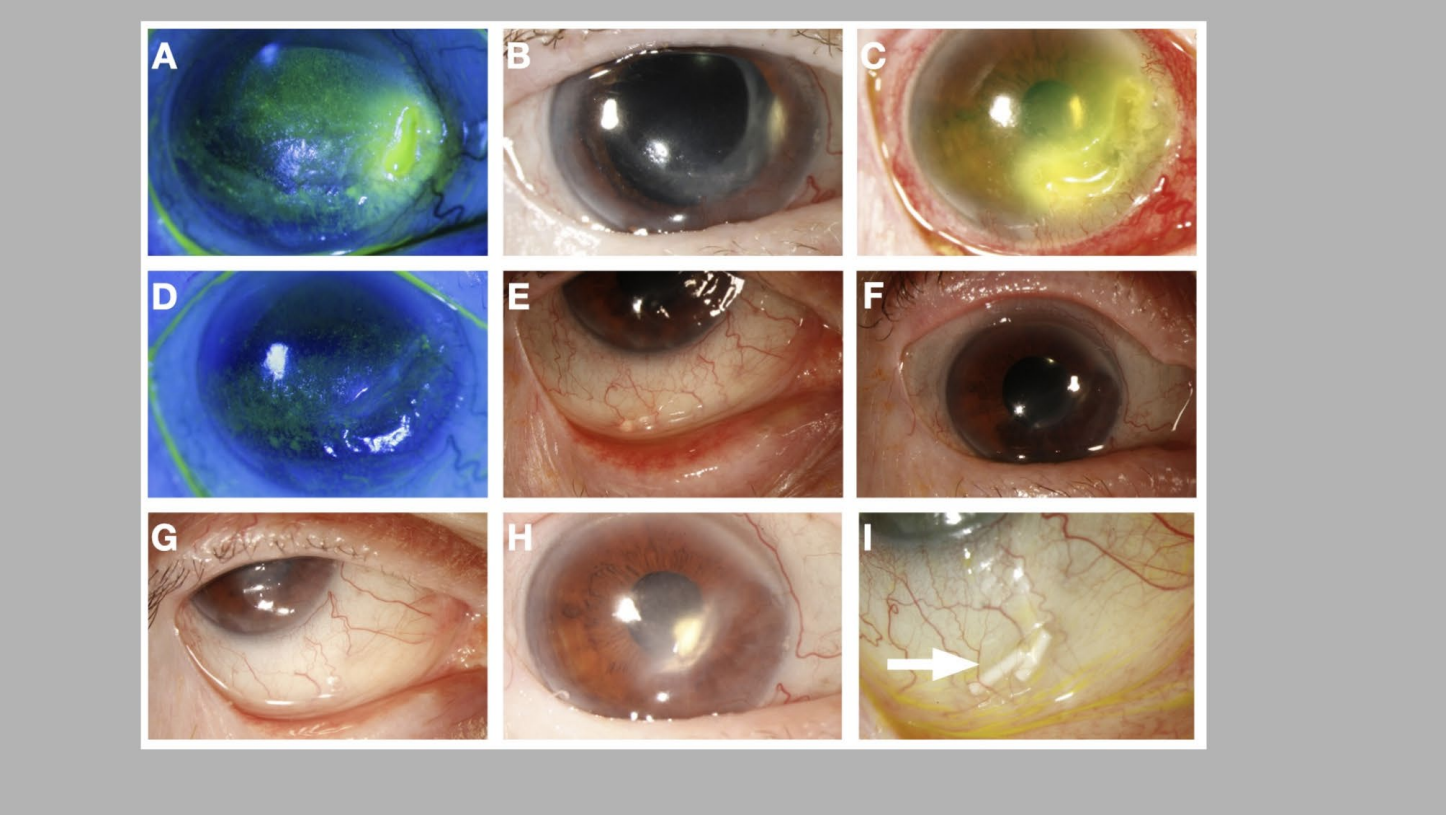
Clinical photographs of Case 3. The slit lamp photos depict the right eye with significant peripheral nasal corneal thinning, about 50%, and a 3 × 1 mm [2] vertical epithelial defect **(A)**. This condition remained stable without recurrent corneal inflammation for approximately 4 months **(B)**. A recurrence of PUK occurred one month after her follow-up appointment, presenting a large nasal, crescent-shaped 5.5 × 3 mm [2] epithelial defect and corneal thinning of up to 85% superiorly **(C)**. The cornea completely re-epithelialized after one month, with resolution of the underlying inflammation **(D)**. Three months later, SDI was administered again **(E**,** F)**. Six months later, another SDI was administered **(G**,** H)**. The DEX implant can be seen in the inferior subconjunctival space following injection **(I)**

**Fig. 4:**
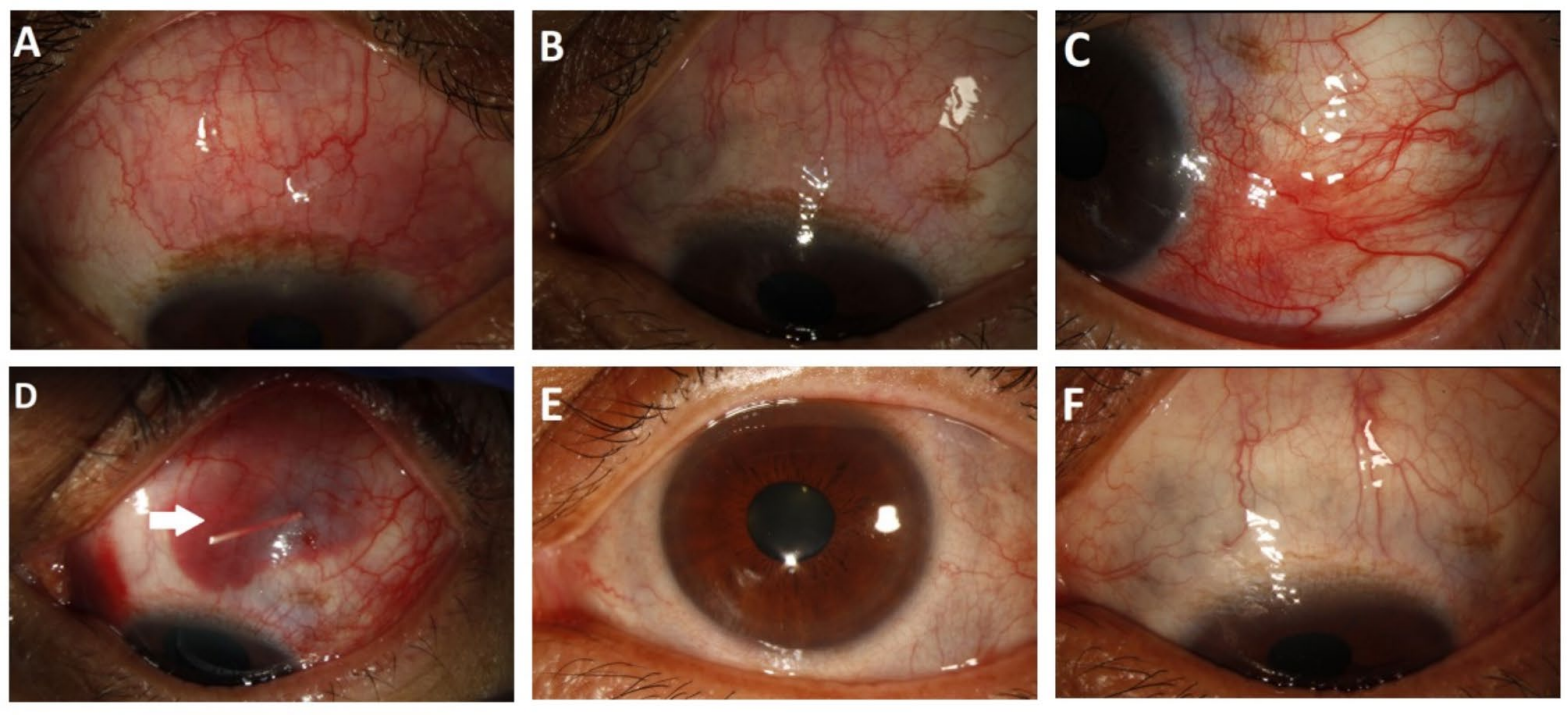
Clinical photographs of Case 4. Slit-lamp examination showed scleritis superiorly and temporally, along with limbal flush in OD **(A)**. Scleral thinning is presented in the superonasal quadrant of OS **(B)**. After 19 cycles of infliximab and IVMP, the patient had recurrent scleritis inferotemporally in OS **(C)**. SDI was present at the superotemporal quadrant with sub-conjunctival hemorrhage **(D)**. Three months after the second injection, scleritis improved significantly, and the SDI was unable to be seen on examination **(E and F)**

An 82-year-old woman presented at a tertiary ophthalmic referral center seeking treatment for PUK in her right eye. Her past medical history includes hypertension, chronic kidney disease, osteoporosis, and anemia, and thyroid cancer diagnosed two years ago. Her ocular history involves thyroid eye disease (TED), cataract surgery in both eyes, and a right temporal tarsorrhaphy following basal cell carcinoma removal from her right lower eyelid. She was treated with a short tapering course of oral prednisone, with doses starting at 30 mg, and valacyclovir 1 g daily, which she was still using at the time of assessment. On examination, her right eye had a BCVA of 20/125, while her left eye had a BCVA of 20/25. IOP measured 10 mmHg in OD and 13 mmHg in OS. Slit-lamp examination revealed significant peripheral nasal corneal thinning of about 50% with a 3 × 1 mm [2] vertical epithelial defect (Fig. 3A), diffuse punctate epithelial keratopathy in OD, and pseudophakia in both eyes. Corneal sensation was markedly decreased in OD but normal in OS. Corneal cultures yielded no bacteria or fungi. Autoimmune markers were mostly negative, except for weakly positive (1:80) anti-nuclear antibody (ANA) titers. Systemic therapy began with 30 mg daily oral prednisone, along with oral methotrexate at 10 mg weekly as steroid-sparing therapy, supplemented with folic acid at 1 mg daily. Repeated corneal cultures returned positive for HSV-1 by PCR. At the 2-month follow-up, the patient reported worsening vision in her eye. The cornea was nearly completely epithelialized, prompting an increase in methotrexate dosage to 12.5 mg weekly. After another month, the cornea was fully epithelialized but with persistent thinning. The patient experienced psychiatric symptoms and multiple falls, leading to a rapid tapering of oral steroids to 10 mg daily. A month later, she was hospitalized for pneumonia and discontinued all medications. Despite being off steroids and methotrexate for four months, she remained stable with no recurrent corneal inflammation (Fig. 3B). However, she experienced a recurrence of PUK one month later, characterized by a large nasal crescent-shaped 5.5 × 3 mm [2] epithelial defect with up to 85% corneal thinning superiorly (Fig. 3C). The patient was not suitable for steroids or steroid-sparing therapy as she and her family declined to resume them. Subsequently, a SDI was offered and accepted as a local therapy option. The procedure involved injecting subconjunctival lidocaine for anesthesia and creating a bleb for the DEX implant injection, which proceeded without complications. A month later, the cornea was fully epithelialized, and inflammation resolved (Fig. 3D**).** The DEX implant was visible in the inferior subconjunctival space (Fig. 3I). Two months later, the cornea remained stable with no recurrence, although the DEX implant had completely disappeared. Approximately three months later, a SDI was administered (Figs. 3E and 4F) due to a flare-up and pain, and again at six months (Fig. 3G and H). Throughout the follow-up periods, there were no signs of scleral thinning or melting, and the intraocular pressure remained normal. The patient was maintained on SDI every 3 months, and remained stable at her last follow up appointment, two years after treatment initiation.

## Case 4

A 51-year-old Indian male was referred to a tertiary ophthalmic referral center for scleritis management. The patient had previously been treated with both oral and topical steroids, resulting in an incomplete response. The patient’s medical history was unremarkable, while the family history revealed that his son had been diagnosed with granulomatosis with polyangiitis (GPA) and was undergoing Rituximab treatment.

At presentation, the patient had BCVA was 20/20 in OD and 20/25 in OS, with IOP of 15 and 14 mmHg, respectively. Slit-lamp examination showed conjunctival injection superiorly and temporally, along with limbal flush in both eyes. Scleral thinning is present in the superonasal quadrant of OS (Fig. 4A and B). Scleritis workup was unremarkable, except for positive RF and slightly elevated erythrocyte sedimentation rate (26 mm/hr). Fundus photography, fluorescein angiography and SD-OCT indicated a normal fundus and posterior scleritis was ruled out with B-scan.

Treatment was started with oral prednisone at 60 mg daily, and mycophenolate mofetil (Cellcept^®^) at 2 g daily. However, scleritis symptoms again recurred after tapering down the oral prednisone dose to 10 mg per day. Due to patient’s preference, (repository corticotropin injection) (RCI, ACTHAR^®^ gel) was started at the dose of 80 U/day three times a week. Several months later, given the continued presence of active scleritis with significant scleral thinning superiorly and significant systemic side effects from oral prednisone use, the treatment approach shifted from RCI to intravenous infliximab 7.5 mg/kg/day once monthly. The biologic was combined with 3 consecutive days of intravenous methylprednisolone (IVMP) at 500 mg per day and a gradual tapering of oral prednisone. After 19 cycles of treatment, the patient’s eyes stabilized, prompting the discontinuation of infliximab and IVMP infusions due to concerns regarding potential side effects. Unfortunately, four months after discontinuation of infliximab and IVMP, the patient experienced left eye redness. Slit-lamp examination revealed recurrent scleritis inferotemporally OS (Fig. 4C) and mild scleritis OD. Considering the persistent active scleritis in OS, a SDI was administered in the superotemporal quadrant OS (Fig. 4D), which demonstrated significant improvement after two months. After five months of inflammation inactivity, a second SDI was administered in the left eye due to a scleritis flare-up. During the follow-up time, the patient remained stable without recurrences of inflammation, and the intraocular pressure remained within the normal range.

## Discussion

The management of scleritis is challenging. Systemic administration of NSAIDs, corticosteroids, immunosuppressive agents, or a combination, is often required for an extended duration [3, 23, 24]. Although it is considered the mainstay of treatment for non-infectious scleritis, compliance and potential side effects may preclude the use of many of these medications [16, 18, 25]. Subconjunctival injections of dexamethasone implant (Ozurdex^®^) could be a viable and safe option in these cases.

Ozurdex^®^ is an FDA-approved intravitreal implant that contains 0.7 mg dexamethasone7 [26]. It uses the Novadur^®^ drug delivery system, which contains a poly D, L sustained lactide-co-glycolide (PLGA) polymer matrix that degrades to lactic acid and glycolic acid7. Such degradation allows for an extended and slow release of the dexamethasone7 [26]. The implant can deliver dexamethasone for up to six months, with a peak effectiveness between 60 and 90 days [26].

In this case series of treatments for non-necrotizing, noninfectious anterior scleritis, we found that for 6 eyes of 4 patients, nearly all experienced improvement in signs and symptoms after SDI with follow-up ranging to 27.5 months. In 2013, Nascimento H, Belfort R, and colleagues employed subconjunctival dexamethasone implant to manage six patients with non-necrotizing anterior scleritis of different types, leading to improvements in symptoms and signs. One recurrence was noted in the 6-month follow-up and the patient was treated with oral steroids [21]. In our series, after one SDI, two of 4 eyes remained free of recurrence for one year. Half of the patients who had adverse effects from systemic medications were off systemic medications at the last follow-up. In addition, in our series, repeated implantations of SDIs (a total of two SDIs in patient #3 and three SDIs in patient #4) were performed without subsequent intraocular hypertension or other ocular adverse events.

The most common side effects of dexamethasone implant reported in patients include subconjunctival hemorrhage and posterior subcapsular cataracts. However, the intravitreal use of the DEX implant caused elevated intraocular pressure in a minority of cases, and therefore, it is important to monitor IOP throughout the effect of the DEX implant [27]. A previous retrospective study supports the use of subconjunctival triamcinolone injection (STI) treatment of non-necrotizing, noninfectious anterior scleritis, with few patients experiencing elevated IOP as a side effect. No cases of scleral melt or necrosis were observed [18].

Of the 6 eyes in this study, 2 eyes had a subconjunctival hemorrhage that resolved spontaneously. Most patients developed cataracts, and 2 eyes required cataract surgery. No eyes in this study developed ocular hypertension, scleral necrosis, or melt. During the mean follow-up period of 27.5 months after SDI, no other adverse events were noted. If needed, the implant can also be safely removed in the outpatient clinic setting, eliminating the need for additional surgical intervention.

The pharmacokinetics of the subconjunctival DEX implant have not been extensively studied. In two previously mentioned studies evaluating the subconjunctival DEX implant, the implant typically completely dissolved in the subconjunctival space within an average of 6 weeks. However, inflammation was generally controlled for an extended period following the resorption of the implant [21, 22]. Similar observations were made in our cases, with the implant being observed at 1 month but disappearing by 3 months post-injection. The majority of our cases exhibited recurrence of the disease. Re-treatment was required for patients with associated systemic disease compared to those with idiopathic anterior scleritis.

Two cases (case 3 and 4) required re-injection of SDI twice, with intervals of 6 and 8 months between each administration. SDI was injected in the superior, superotemporal, and inferior quadrants, with no observed differences among them. Prior to SDI administration, patients had been prescribed one to three medications, including oral steroids, antimetabolites, and biologics. Although there is limited data and studies on the use of SDI, our case series demonstrates that Ozurdex^®^ implantation via the subconjunctival route holds promise as a therapeutic option for scleritis, even in cases that have been recurrent or refractory to systemic biologics.

Valizadeh et al. reported the resolution of treatment-resistant PUK through successive SDI and STI injections after the failure of systemic therapy with steroids, azathioprine, and rituximab [28]. In our cases, due to the patient’s inability to tolerate various systemic treatments, we opted to administer the DEX implant rather than TA for two reasons: the reported lower incidence of ocular hypertension [29] and the ease of removal in case of adverse events. Such advantages underscores the potential significance of subconjunctival steroids in managing PUK (case 3), particularly in cases where systemic immunosuppression is either intolerable or ineffective.

SDI represents a valuable adjunctive therapy in the management of persistent scleritis, offering an alternative for patients with poor compliance or significant comorbidities. Further research and clinical trials are warranted to elucidate its efficacy, safety profile, and optimal treatment protocols in this challenging ocular condition.

## Conclusion

In summary, subconjunctival injection of Ozurdex was safely and effectively used for the local treatment of refractory non-necrotizing anterior scleritis. The main advantage of the use of SDI is in those with poor compliance or those with contraindications to systemic management. The use of local treatment may also avoid the masking of systemic diseases, allowing for proper management of the underlying systemic ailments. Retreatment is needed in select cases and has been well tolerated. Further studies are required to fully elucidate its efficacy and optimal use in the treatment of scleritis.

**Table 1:** Summary of demographics and clinical course of patients

Patient	Age (years)	Gender	Scleritis type (all anterior non-necrotizing)	Etiology	Concomitant medications	Recurrence
1	60	Female	Diffuse	Rheumatoid arthritis	Oral prednisone, methotrexate, leflunomide, azathioprine, adalimumab, etanercept, abatacept, certolizumab, rituximab, and tofacitinib, cyclophosphamide	+
2	36	Male	Diffuse	Granulomatosis with polyangiitis	Oral prednisone, Rituximab, mycophenolate mofetil, tofacitinib, cyclophospahmide, daratumumab	-
3	82	Female	Diffuse	Idiopathic	Oral prednisone	+
4	51	Male	Diffuse	Idiopathic	Oral prednisone, mycophenolate mofetil, Rituximab, ACHTAR gel, IVMP, Infliximab	+

## Data Availability

The datasets generated and analysed is available upon reasonable request and in compliance with local data protection policy.
